# Prevalence and Risk Factors of Postprocedure Depression in Patients with Atrial Fibrillation after Radiofrequency Ablation

**DOI:** 10.1155/2023/4635336

**Published:** 2023-11-07

**Authors:** Mingli Du, Tieniu Cheng, Yutong Ye, Yong Wei

**Affiliations:** ^1^Department of Nursing, Shanghai General Hospital, Shanghai Jiao Tong University School of Medicine, Shanghai, China; ^2^Department of Cardiology, Shanghai General Hospital, Shanghai Jiao Tong University School of Medicine, Shanghai, China; ^3^Department of Cardiology, Tongling People's Hospital, Tongling, Anhui, China

## Abstract

**Background:**

Recent studies have shown a bidirectional relationship between atrial fibrillation (AF) and psychological depression. However, little is known about the prevalence of postprocedure depression (PPD) in patients with AF at the time of radiofrequency (RF) ablation.

**Objective:**

To describe the prevalence and identify risk factors for PPD.

**Methods:**

This was a prospective cohort study, including 428 AF patients who were willing to undergo the first catheter ablation in our hospital from 1^st^ April to 30^th^ December 2019. The primary outcome was PPD, which was determined by Hospital Anxiety and Depression Scale-Depression.

**Results:**

The prevalence of PPD was 16.8% (72/428) in our cohort, without difference between men (16.0%, 41/256) and women (18.0%, 31/172) (*P* = 0.586) but with a great difference among different age groups (*P* = 0.016). On the univariable binary logistic regression analysis, age, a history of coronary heart disease, Observer's Assessment of Alertness/Sedation (OAA/S) score when ablating at the specific position, and OAA/S score when pulling out the catheter sheath were associated with PPD. Subsequent multivariable logistic regression analysis indicated only age (OR 0.96, 95% CI: 0.94–0.99, *P* <  0.01) and OAA/S score when ablating at the specific position (OR 0.58, 95% CI: 0.39–0.88, *P* = 0.01) were independently associated with PPD.

**Conclusion:**

PPD is common in patients with AF after RF ablation. Younger age and lower OAA/S score when ablating at the specific position are its most significant risk factors. Intensive management of sedation may be of great importance for reducing PPD. This trial is registered with the Chinese Clinical Trial Registry (ChiCTR2200057810).

## 1. Introduction

Atrial fibrillation (AF) is a common arrhythmia, which usually causes serious complications such as stroke and heart failure. Some studies have shown that early rhythm control for AF patients can significantly improve the prognosis [1, 2]. Catheter ablation has been recommended as the first-line rhythm-control strategy by guidelines for AF management [3, 4]. In China, most radiofrequency (RF) ablations of AF are performed under local anesthesia with conscious analgesia and sedation. Patients might feel the pain during ablation and get discomfort for being on the table for a long time to avoid movements. So, AF patients may suffer from psychological distress in forms of depression after ablation.

A bidirectional relationship between AF and psychological depression may exist. Patients with AF were prone to have depressive symptoms and anxiety [5, 6]. Meanwhile, a high burden of psychologic distress seemed to be an independent risk factor for AF, acting as a trigger, creating an arrhythmogenic substrate, and modulating the autonomic nervous system [7, 8]. Psychological distress not only causes the symptoms of AF more serious [9] but also increases the recurrence risk of AF after circumferential pulmonary vein ablation [10, 11]. So, it is important to find the causes of postprocedure depression (PPD) for making strategies to prevent it. A recent survey from AF patient's preparation for catheter ablation showed that most of them felt anxiety and depression before the operation [12]. However, little is known about PPD. The purpose of this study was to investigate the prevalence of PPD in patients undergoing AF ablation with conscious analgesia and sedation, accordingly exploring the risk factors of PPD.

## 2. Methods

### 2.1. Ethics Statement

This study had the ethical approval from the Ethics Commission of the Institutional Review Board of Shanghai General Hospital, Shanghai Jiao Tong University School of Medicine, Shanghai, China. It was conducted in accordance with the Helsinki Declaration. All participants were informed of the nature and objectives of the study. Written informed consent was obtained from each enrolled subject. The trial was registered in the Chinese Clinical Trial Registry (ChiCTR2200057810).

### 2.2. Study Design and Patient Selection

This is a prospective cohort study conducted in Shanghai General Hospital, Shanghai Jiao Tong University School of Medicine, Shanghai, China. The convenience sample method was used to select the patients who were hospitalized for AF ablation in our hospital from 1^st^ April to 30^th^ December 2019. The inclusion criteria were as follows: (1) age ≥20 years, (2) diagnosis of nonvalvular AF, (3) no previous ablation of AF, (4) acceptance of RF ablation with conscious sedation, and (5) with good communication skills and reading comprehension. The exclusion criteria were as follows: (1) patients who had suffered nervous system diseases such as stroke, dementia, and Parkinson disease and (2) patients with severe mental illness.

### 2.3. Assessment and Data Collection

The research team developed a questionnaire on general information, including hospitalization number, date of birth, gender, weight, education, marital status, long-term residence, occupational status, way of caring, payment manner of medical expenses, subtype of AF, symptom grade, comorbidities, and procedure time.

The Hospital Anxiety and Depression Scale (HADS) was selected to screen perioperative anxiety and depression in patients undergoing AF ablation. It has 2 subdimensions, which are Anxiety (HADS-A) and Depression (HADS-D). Each subdimension consists of 7 items. Each question has 4 options, whose scores vary from 0 to 3. Scores for HADS-A and HADS-D are separately calculated and evaluated. If the score obtained from each subdimension is greater than or equal to 8, it indicates an abnormal situation of anxiety and depression, respectively. Cronbach's alpha value was found to be 0.753 for the anxiety subdimension and 0.764 for the depression subdimension in this study.

The Wong–Baker scale was used for the assessment of intraoperative pain. It was a 10-point scoring system for “0” representing painlessness and “10” representing unbearably severe pain. The pain intensity was divided into three degrees: mild (scoring 1–3), moderate (scoring 4–6), and severe (scoring 7–10). It was repeatedly assessed at multiple key time points during the procedure.

The Observer's Assessment of Alertness/Sedation (OAA/S) scale was used to measure the level of alertness in subjects who were sedated. The details of OAA/S were previously reported, with the cumulative scores of the responsiveness, speech, facial expression, and eyes component [13].

The questionnaires of the general data and the HADS were filled in by patients under the guidance of the data collectors, who explained details of the questionnaire to patients and collected the questionnaires on the spot. The intraoperative questionnaire was filled in by the data collector after they communicated with the patients and evaluated the patient's condition.

Data collectors started their work only after training and passing the examination. The training contents included study interpretation, practical skills of data collection, issues with survey form-filling, pain assessment, definition of key time points, and other aspects. After the training, all data collectors were informed to independently collect data from the same patient. Taking the skilled researcher who provided the training as reference, it constituted failure when the overall difference of the contents filled in by the data collector exceeding 5% and the pain score exceeding 1 point.

### 2.4. Ablation Procedure

Before the ablation procedure, patients' baseline information including a clinical assessment, a transesophageal echocardiogram, a 12-lead electrocardiogram (ECG), and blood tests were collected. Left atrial access was achieved by the transseptal approach. Catheter navigation, mapping, and ablation were guided by CARTO 3 (Biosense Webster, Diamond Bar, CA, USA) or EnSite Precision system (Abbott, St. Paul, MN, USA). The ablation protocol consisted of circumferential pulmonary vein (PV) antrum isolation and additional ablation recommended in the guidelines, including ablation of non-PV triggers and the induced or spontaneously developing atrial tachycardias, as well as limited linear ablation and low-voltage-area ablation decided by the physician. Bidirectional block across the linear lesion is the endpoint of the linear ablation. Ablation was performed by delivering RF energy with the ablation catheter, and the energy setting was 35 W.

### 2.5. Conscious Analgesia and Sedation

Conscious analgesia/sedation was achieved by intravenous administration of loading doses of 0.8 *μ*g/kg fentanyl and 0.01 mg/kg midazolam, followed by continuous infusion of 1 *μ*g/kg/h fentanyl and 0.01 mg/kg/h midazolam with a syringe pump. If the patients felt pain during ablation, the maintenance dose of fentanyl was increased to less than 2 *μ*g/kg/h. Another 0.5–1 mg midazolam was added if the pain was not relieved by the adjustment of fentanyl.

### 2.6. Outcomes

The main outcome of the study was the incidence of PPD during the 3-day follow-up after RF ablation. Depression was assessed using HADS-D, of which a score of 8 or above indicated possible psychological distress in the form of depression.

### 2.7. Statistical Analysis

Continuous variables with normal distribution were presented as mean (standard deviation) and the others as median (interquartile range). Categorical variables were presented as number (percentage). The independent samples *t*-test was used to compare normally distributed continuous variables. For the comparison of nonnormally distributed data, the Mann–Whitney *U* test was performed. The chi-squared test was used to compare the categorical variables between the two groups. The Wilcoxon rank sum test was used for ranked data between groups. The univariable and multivariable binary logistic regression analyses (forward stepwise: likelihood ratio) were applied to identify risk factors for PPD. All statistical analyses were performed with SPSS 13.0. Two-tailed *P* value less than 0.05 was considered of statistical significance.

## 3. Results

### 3.1. Baseline Characteristics of the Enrolled Subjects

A total of 492 questionnaires were returned, and 64 questionnaires were excluded for incomplete data in filling. Finally, 428 questionnaires were involved in the analysis, yielding a useable response rate of 87.0%. The enrolled 428 subjects included 256 males and 172 females, with the median age of 67 (61–73) years old. The baseline characteristics are presented in Table 1. Compared with patients without PPD, patients with PPD tended to be younger and had more frequent CHD and hypertension, higher preoperative depression scores, greater X-ray exposure, and lower OAA/S scores when ablating at the specific position and when pulling out the catheter sheath.

### 3.2. The Prevalence of PPD in Patients with AF after RF Ablation

**Figure 1:**
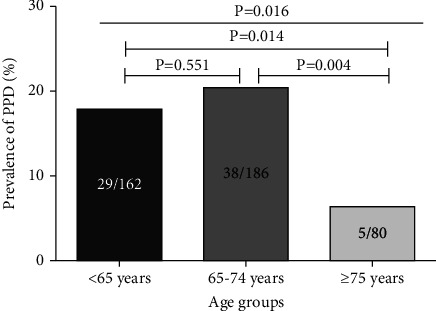
The prevalence of postprocedure depression (PPD) among the three age groups (<65, 65–74, and ≥75 years).

The prevalence of PPD was 16.8% (72/428) in our cohort, without difference between men (16.0%, 41/256) and women (18.0%, 31/172) (*P* = 0.586). However, a great difference in PPD prevalence was found among the three age groups, such as <65, 65–74, and ≥75 years (*P* = 0.016, Figure 1).

### 3.3. The Risk Factors of PPD in Patients with AF after RF Ablation

On the univariable binary logistic regression analysis, only age, CHD, OAA/S score when ablating at the specific position, and OAA/S score when pulling out the catheter sheath were associated with PPD (Table 2). They were selected for multivariable logistic regression analysis, and we found only age (OR 0.96, 95% CI: 0.94–0.99, *P*  <  0.01) and OAA/S score when ablating at the specific position (OR 0.58, 95% CI: 0.39–0.88, *P* = 0.01) were independently associated with PPD (Table 3).

## 4. Discussion

This prospective cohort study demonstrated that PPD was common in patients with AF after RF ablation with conscious analgesia and sedation. Younger age and lower OAA/S score when ablating at the specific position were its most significant risk factors. Intensive management of OAA/S score when ablating at the specific position may be of great importance for reducing PPD.

Catheter ablation has been increasingly selected as the first-line strategy of rhythm control for AF [3, 4]. Patients who undergo RF ablation of AF with conscious sedation often feel pain and nervous during the procedure. So, some patients may suffer from psychological distress after ablation. An increasing number of studies have examined a bidirectional association between depressive symptoms and AF [14, 15]. Psychological stress can be elicited by AF episodes and might also predispose to AF occurrence. A meta-analysis of cohort studies that evaluated the association between depression and AF recurrence showed depression was an independent risk factor of AF recurrence after catheter ablation [16]. Therefore, the screening of depression should be taken seriously after RF ablation. The prevalence of depression in patients with AF before catheter ablation has been reported as high as 17–43% [17–19]. It varied greatly for various symptom-based tools applied on depression assessment, such as Self-Rating Depression Scale (SDS), Beck Depression Inventory (BDI), Center for Epidemiologic Studies Depression (CESD) scale, Kellner Symptom Psychometric Questionnaire (KSPQ), and mental health inventory-5 (MHI-5). In this study, only HADS-D was applied to measure the depressive symptoms. Our data showed that 19.4% of AF patients who were willing to undergo RF ablation with conscious sedation suffered from preprocedure depression. Undetected depressive mood may undermine the treatment effects after RF ablation. So, it is essential to monitor the depressive symptoms during the perioperative period. We found the prevalence of depression was 16.8% after ablation, with no difference compared with the prevalence of preoperative depression.

As shown by the results of this study, the incidence of PPD is relatively high. Careful psychological evaluation and appropriate management are required to improve patients' quality of life after RF ablation. Thus, it is important to clarify the risk factors of PPD, so as to explore treatment strategies. We evaluated the associations of individual characteristics, comorbidities, AF-related factors, pain severity during ablation, and psychological distress with PPD. It showed that younger age and lower OAA/S score when ablating at the specific position were independently associated with PPD. The relationship between age and depression in patients with AF is controversial, for some studies indicated that younger age was associated with higher levels of psychological distress [20, 21], while another study showed that elderly AF patients were more likely to suffer from depression [22]. In addition, Koleck et al. demonstrated that age was not associated with depressive symptoms among patients with AF at the time of cardioversion or ablation [17]. Our data showed patients who suffered from PPD were younger than those without PPD and multivariate analysis indicated younger age was an independent risk factor of PPD.

Considering that severe pain might occur during the ablation of the posterior wall of the left atrium, we usually increase the dose of fentanyl for optimal pain relief and administer 0.5–1.0 mg of midazolam to temporarily enhance sedation. Subjectively, it was believed that good analgesia and deeper sedation could alleviate the patient's pain during ablation, thereby reducing the occurrence of PPD. But objectively, compared with patients without PPD, patients with PPD had lower OAA/S score when ablating at the specific position. It implies that deeper sedation works as an independent risk factor for PPD. Therefore, we demonstrated that excessive conscious sedation during the ablation at the specific position might increase the occurrence of PPD. Several explanations may account for this, such as adverse reaction of the sedative, psychological impairment, or poor management of pain. We primarily supposed that preoperative depression might aggravate PPD. A study suggested that worse preoperative depression and postoperative pain predicted higher postoperative depression [23]. However, actually in our sample, no significant relationship was found between PPD and preoperation psychological distress. Preoperative anxiety or depression might mainly come from the worry about the procedure risks. But PPD might mainly come from concerns about the effectiveness of the invasive treatment. So, they worked independently.

This study has a great clinical implication for the positive correlation was seen with younger age and lower OAA/S score when ablating at the specific position, which is an indicator of pain threshold. So, we may argue that this perception could be related to brain involution that is indirectly related with younger age. Indeed, as recently showed in one study [24] that AF is often triggered by synchronized supraventricular tachycardias, which are easier, less time-consuming, less risky, and less painful to ablate. Moreover, this phenomenon is more common in younger people, who represent the population at higher risk of developing PPD according to our study. Therefore, in order to reduce PPD especially in young AF patients, it is important to search wisely any trigger arrhythmia, instead of submitting them to longer and extensive ablation such as substrate modification and rotor ablation.

### 4.1. Limitations

The present study has certain limitations. First, it is a single-center observational study and included a small sample size. Therefore, the findings of this study need to be validated further in larger populations. Second, the follow-up period was relatively short. We only evaluated PPD before discharge and did not continue to follow up for a longer period of three months or one year after ablation. Long follow-up periods might reveal additional psychological changes. Third, loss of long-term follow-up data prevented us from determining if levels of PPD symptoms were associated with AF recurrence after catheter ablation.

## 5. Conclusions

In summary, this study finds that PPD is common in patients with AF after RF ablation. Younger age and lower OAA/S score when ablating at the specific position are its most significant risk factors. Intensive management of sedation may be of great importance to reduce PPD. Additional studies on assessment, prediction, and treatment of PPD in patients with AF after RF ablation are warranted.

## Figures and Tables

**Table 1:** Baseline characteristics.

	Total, *N* = 428	Without PPD, *N* = 356	With PPD, *N* = 72	*P* values
Age, years old	67.0 (61.0–73.0)	67.5 (62.0–73.0)	66.5 (59.0–71.0)	0.04
Male, *n* (%)	256 (59.8)	215 (60.4)	41 (56.9)	0.59
Married, *n* (%)	400 (93.5)	333 (93.5)	67 (93.1)	1.00
Occupation, *n* (%)				
On the job	68 (15.9)	51 (14.3)	17 (23.6)	0.13
Retire	336 (78.5)	286 (80.3)	50 (69.4)	
Others	24 (5.6)	19 (5.3)	5 (6.9)	
Residence, *n* (%)				
Town	102 (23.8)	87 (24.4)	15 (20.8)	0.51
City	326 (76.2)	269 (75.6)	57 (79.2)	
Education, *n* (%)				
Postgraduate	8 (1.9)	5 (1.4)	3 (4.2)	0.33
Bachelor's degree	100 (23.4)	80 (22.5)	20 (27.8)	
Secondary school	250 (58.4)	213 (59.8)	37 (51.4)	
Primary school	70 (16.4)	58(16.3)	12 (16.7)	
Body weight, *n* (%)	68.0 (60.0–75.8)	67.0 (59.3–75.0)	70.0 (63.5–80.0)	0.05
Hypertension, *n* (%)	227 (53.0)	182 (51.1)	45 (62.5)	0.08
Diabetes	72 (16.8)	58 (16.30)	14 (19.4)	0.51
CHD, *n* (%)	69 (16.1)	51 (14.3)	18 (25.0)	0.03
CVD, *n* (%)	29 (6.8)	27 (7.6)	2 (2.8)	0.22
Smoking, *n* (%)	54 (12.6)	43 (12.1)	11 (15.3)	0.46
Drinking, *n* (%)	34 (7.9)	29 (8.1)	5 (6.9)	0.73
Payment by medical insurance, *n* (%)	336 (78.5)	280 (78.7)	56 (77.8)	0.87
Symptom grade				
EHRA I	127 (29.7)	107 (30.1)	20 (27.8)	0.47
EHRA II	193 (45.2)	162 (45.6)	31 (43.1)	
EHRA III	104 (24.4)	83 (23.4)	21 (29.2)	
EHRA IV	3 (0.7)	3 (0.8)	0 (0.0)	
Frequency of AF attack, *n* (%)				
Occasionally	208 (48.6)	167 (46.9)	41 (56.9)	0.20
Sometimes	110 (25.7)	96 (27.0)	14 (19.4)	
Frequently	110 (25.7)	93 (26.1)	17 (23.6)	
Ways of caring				
No accompanying	32 (7.5)	25 (7.0)	7 (9.7)	0.17
Nanny's	8 (1.9)	8 (2.2)	0 (0.0)	
Family member's	388 (90.7)	323 (90.7)	65 (90.3)	
Preprocedure ASA grade				
I	110 (25.7)	89 (25.0)	21 (29.2)	0.44
II	297 (69.4)	249 (69.9)	48 (66.7)	
III	21 (4.9)	18 (5.1)	3 (4.2)	
Intraoperative ECV, times	1 (0-1)	1 (0-1)	1 (0-1)	0.88
Complication, *n* (%)	14 (3.3)	11 (3.1)	3 (4.2)	0.92
Procedure time, minutes	243.0 (200.0–292.0)	243.0 (200.0–292.0)	241.5 (210.0–292.0)	0.60
Ablation time, seconds	3369.0 (2543.3–4164.0)	3360.5 (2592.5–4200.0)	3420.0 (2472.8–4125.0)	0.69
X-ray time, seconds	448.0 (318.5–628.5)	451.0(323.0–62 8.5)	422.0(317.0–61 3.8)	0.61
X-ray dose, mGy	41.0 (29.0–63.0)	39.5 (28.3–60.0)	47.0 (31.0–71.5)	0.04
Subtypes of AF, *n* (%)				
PAF	260 (60.7)	211 (59.3)	49 (68.1)	0.16
PsAF	168 (39.3)	145 (40.7)	23 (31.9)	
Mapping system				
Carto	342 (79.9)	283 (79.5)	59 (81.9)	0.64
Rhythmia	86 (20.1)	73 (20.5)	13 (18.1)	
Preprocedure anxiety score	7 (5–9)	7 (5–9)	7 (5–9)	0.64
Preprocedure anxiety, *n* (%)	143 (33.4)	123 (34.6)	20 (27.8)	0.27
Preprocedure depression score	6.0 (3.0–7.0)	6.0 (3.0–7.0)	6.5 (5.0–7.0)	0.04
Preprocedure depression, *n* (%)	83 (19.4)	73 (20.5)	10 (13.9)	0.20
CD-RISC	63 (60–65)	63 (60–65)	64 (60–65)	0.91
Strength	21 (20–23)	21 (20–23)	21 (19–22)	0.16
Optimism	11 (10–12)	11 (10–12)	11 (10–12)	0.17
Tenacity	30 (29–33)	30 (29–33)	31 (29–33)	0.45
OAA/S score				
Before giving sedative	5 (5-5)	5 (5-5)	5 (5-5)	0.50
During ablation	5 (5-5)	5 (5-5)	5 (4-5)	0.52
When ablating at the specific position	5 (4-5)	5 (4-5)	4 (4-5)	0.03
When pulling out the catheter sheath	5 (5-5)	5 (5-5)	5 (5-5)	0.027
Postprocedure	5 (5-5)	5 (5-5)	5 (5-5)	0.17
Pain scores				
Before giving sedative	2 (2-3)	2 (2-3)	2 (2-2)	0.60
During ablation	4 (3–5)	4 (3–5)	4 (3–5)	0.49
When ablating at the specific position^*∗*^	5 (4–6)	5 (4–6)	5 (4–6)	0.96
When pulling out the catheter sheath	3 (2–4)	3 (2–4)	2 (2–3.8)	0.50
Postprocedure	2 (1-2)	2 (1-2)	1 (1-2)	0.41

PPD, postprocedure depression; AF, atrial fibrillation; CHD, coronary heart disease; CVD, cerebrovascular disease; EHRA, European Heart Rhythm Association; ECV, electrocardioversion; OAA/S, observer's assessment of alertness/sedation scale; PAF, paroxysmal atrial fibrillation; PsAF, persistent atrial fibrillation; CD-RISC, Connor–Davidson resilience scale; CAS, conscious analgesia and sedation. ^*∗*^The specific position was defined as the ablation site of the posterior and inferior wall of the left atrium.

**Table 2:** Univariable binary logistic regression analysis.

Variables	*Univariable logistic regression*	
	OR (95% CI)	*P*
Age	0.97 (0.94–0.99)	0.01
Male	0.87 (0.52–1.45)	0.59
Married	0.93 (0.34–2.52)	0.88
Occupation	0.69 (0.4–1.2)	0.19
Residence	0.81 (0.44–1.51)	0.51
Education	0.8 (0.55–1.16)	0.23
Body weight	1.02 (0.99–1.04)	0.18
Hypertension	1.59 (0.95–2.68)	0.08
Diabetes	1.24 (0.65–2.37)	0.52
CHD	1.99 (1.08–3.67)	0.03
CVD	0.35 (0.08–1.5)	0.16
Smoking	1.31 (0.64–2.69)	0.46
Drinking	0.84 (0.31–2.25)	0.73
Payment	0.95 (0.52–1.75)	0.87
Symptom grade	1.11 (0.8–1.56)	0.53
Frequency of AF attack	0.83 (0.61–1.14)	0.24
Ways of caring	0.90 (0.58–1.41)	0.65
Preoperative ASA grade	0.83 (0.51–1.35)	0.45
Intraoperative ECV	1.07 (0.78–1.46)	0.70
Complication	1.36 (0.37–5.02)	0.64
Procedure time	1.00 (1.00–1.01)	0.60
Ablation time	1.00 (1.00–1.00)	0.97
X-ray time	1.00 (1.00–1.00)	0.69
X-ray, mGly	1.01 (1.00–1.01)	0.10
PAF	1.46 (0.85–2.51)	0.17
Mapping system	1.17 (0.61–2.25)	0.64
Preoperative anxiety score	1.01 (0.94–1.08)	0.75
Preoperative anxiety	0.73 (0.42–1.28)	0.27
Preoperative depression score	1.04 (0.97–1.11)	0.31
Preoperative depression	0.63 (0.31–1.28)	0.20
CD-RISC	1.01 (0.95–1.07)	0.84
Strength	0.96 (0.87–1.06)	0.42
Optimism	1.08 (0.95–1.24)	0.25
Tenacity	1.01 (0.94–1.09)	0.80
OAA/S		
Before giving sedative	0.73 (0.28–1.86)	0.50
During ablation	1.08 (0.95–1.24)	0.23
When ablating at the specific position	^∗^0.61 (0.41–0.91)	0.02
When pulling out the catheter sheath	0.54 (0.32–0.94)	0.03
Postprocedure	0.58 (0.3–1.1)	0.10
Pain scores		
Before giving sedative	0.96 (0.69–1.32)	0.79
During ablation	0.93 (0.76–1.14)	0.50
When ablating at the specific position	^∗^0.98 (0.82–1.18)	0.85
When pulling out the catheter sheath	0.93 (0.74–1.17)	0.55
Postprocedure	0.83 (0.61–1.14)	0.25

OR, odds ratio; CI, confidence interval; CHD, coronary heart disease; CVD, cerebrovascular disease; ECV, electrocardioversion; OAA/S, observer's assessment of alertness/sedation scale; PAF, paroxysmal atrial fibrillation; CD-RISC, Connor–Davidson resilience scale; CAS, conscious analgesia and sedation. ^*∗*^The specific position was defined as the ablation site of the posterior and inferior wall of the left atrium.

**Table 3:** Multivariable logistic regression analysis of selected variables.

Selected variables	Enter		*Forward stepwise: LR*	
	OR (95% CI)	*P*	OR (95% CI)	*P*
Age	0.97 (0.94–0.99)	0.01	0.96 (0.94–0.99)	0.01
CHD	1.83 (0.98–3.42)	0.06	—	—
OAA/S when ablating at the specific position	0.68 (0.43–1.07)	0.10	0.58 (0.39–0.88)	0.01
OAA/S when pulling out the catheter sheath	0.64 (0.35–1.18)	0.16	—	—

OR, odds ratio; CI, confidence interval; LR, likelihood ratio; CHD, coronary heart disease; OAA/S, observer's assessment of alertness/sedation scale.

## Data Availability

No underlying data were collected or produced in this study.
